# Stochastic Binding Dynamics of a Photoswitchable Single Supramolecular Complex

**DOI:** 10.1002/advs.202200022

**Published:** 2022-03-02

**Authors:** Dingkai Su, Shuyao Zhou, Hiroshi Masai, Zihao Liu, Ce Zhou, Chen Yang, Zhizhou Li, Susumu Tsuda, Zhirong Liu, Jun Terao, Xuefeng Guo

**Affiliations:** ^1^ Beijing National Laboratory for Molecular Sciences National Biomedical Imaging Center College of Chemistry and Molecular Engineering Peking University Beijing 100871 P. R. China; ^2^ Department of Basic Science Graduate School of Arts and Sciences The University of Tokyo Tokyo 153‐8902 Japan; ^3^ Department of Chemistry Osaka Dental University Osaka 573‐1121 Japan; ^4^ Center of Single‐Molecule Sciences Institute of Modern Optics Frontiers Science Center for New Organic Matter College of Electronic Information and Optical Engineering Nankai University 38 Tongyan Road, Jinnan District Tianjin 300350 P. R. China

**Keywords:** conductance, host‐guest interaction, photoswitch, single molecule, supramolecular electronics

## Abstract

In this work, a real‐time precise electrical method to directly monitor the stochastic binding dynamics of a single supramolecule based on the host‐guest interaction between a cyclodextrin and an azo compound is reported. Different intermolecular binding states during the binding process are distinguished by conductance signals detected from graphene‐molecule‐graphene single‐molecule junctions. In combination with theoretical calculations, the reciprocating and unidirectional motions in the *trans* form as well as the restrained reciprocating motion in the *cis* form due to the steric hindrance is observed, which could be reversibly switched by visible and UV irradiation. The integration of individual supramolecules into nanocircuits not only offers a facile and effective strategy to probe the dynamic process of supramolecular systems, but also paves the way to construct functional molecular devices toward real applications such as switches, sensors, and logic devices.

## Graphical abstract

In‐situ real‐time electrical recordings of a photoswitchable shuttling process are realized through graphene‐molecule‐graphene single‐molecule junctions for the first time. Different motion phenomena hidden in the stochastic motion are observed directly and can be switched through irradiation of visible and UV light.

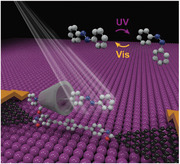

## 1 Introduction

Supramolecules are the organized entities of two or more molecular components held together by non‐covalent intermolecular forces such as host–guest interactions, hydrogen bonds and *π*–*π* interactions.^[^ ^1^, ^2^, ^3^ ^]^ Due to the lability of non‐covalent interactions, supramolecular entities are by nature constitutionally dynamic, giving rise to many practical applications in sensing,^[^ ^4^, ^5^ ^]^ molecular imaging^[^ ^6^, ^7^ ^]^ and biological processes.^[^ ^8^, ^9^, ^10^ ^]^ In most cases, researchers characterize these non‐covalent interactions through traditional thermodynamic techniques like NMR^[^ ^11^ ^]^ and fluorescence spectroscopy,^[^ ^12^, ^13^ ^]^ which study the statistical average behavior of a large number of molecules at the same time and provide invaluable structural insights, but shield the subtle dynamic character of a single molecule such as the time‐sequence relations and the hidden intermediates.

Single‐supramolecule electronics offers an excellent platform to probe the detailed dynamic properties of an individual supramolecule because of its high resolution and sensitivity both spatially and temporally.^[^ ^14^, ^15^, ^16^ ^]^ The principle of the method is to transduce the chemical information into the detectable changes in the molecular conductance.^[^ ^17^, ^18^, ^19^ ^]^ In the previous work from our group, graphene‐molecule‐graphene single‐molecule junctions (GMG‐SMJs) have been proved to be a robust electrical detection platform for tracking submolecular changes at the single‐event level, for example, single‐molecule chemical reactions,^[^ ^20^, ^21^, ^22^, ^23^ ^]^ stereoelectronic effect^[^ ^24^ ^]^ and hydrogen‐bonding dynamics.^[^ ^25^ ^]^

**Figure 1:**
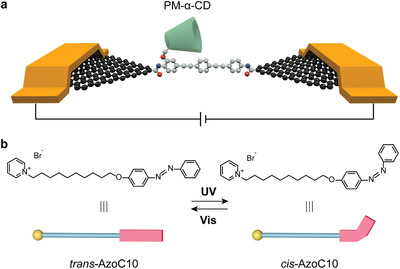
Schematic illustration of a photoswitchable single supramolecule. a) Device structure of GMG‐SMJs featuring a PM‐*α*‐CD covalently bonded to graphene electrodes through amide bonds. b) Molecular structures and schematic representation of AzoC10 that can isomerize reversibly between *trans* and *cis* forms upon irradiation with visible and UV lights. The yellow ball represents the pyridinium headgroup and the blue string represents the long alkyl chain of AzoC10. The red bar represents the azobenzene moiety, with the straight one for the *trans* isomer and the bent one for the *cis* isomer.

In this work, we demonstrated the in‐situ, real‐time electrical detection of the stochastic motion of a photoswitchable single supramolecule by using GMG‐SMJs. We designed a pseudorotaxane structure with permethylated‐*α*‐cyclodextrin (PM‐*α*‐CD) as a cavity and 1‐[10‐(4‐phenylazophenoxy)decyl] pyridinium bromide (AzoC10) as an axle. As shown in **Figure** **1**a, a rigid conjugated molecular wire was connected to graphene electrodes through covalent bonds as a stable and highly conductive channel. A PM‐*α*‐CD was immobilized as the side‐group of the wire and its different binding states could affect the conductance of the channel, similar to the gate regulation in three‐terminal devices. There are two reasons why *α*‐CD was permethylated. Firstly, a dehydration‐condensation reaction was carried out for molecular connection. It is possible that carboxyl groups at the edge of graphene electrodes react with hydroxyl groups of *α*‐CD rather than amino groups of the oligo phenylene ethynylene (OPE) molecule, thus reducing the connection yield. Secondly, intramolecular hydrogen bonds on the outer surface of *α*‐CD could be eliminated in this way, which contributes to removing the interference of irrelevant processes and improving the signal‐to‐noise ratio. The amphiphilic AzoC10 consists of three parts, the hydrophilic pyridinium unit, the hydrophobic alkyl chain and the azobenzene moiety.^[^ ^26^, ^27^ ^]^ Irradiation can be used as convenient external stimuli to adjust the conductance of molecular devices since isomerization can be introduced by irradiation, and examples include diarylethene,^[^ ^28^, ^29^ ^]^ spiropyrans,^[^ ^30^, ^31^ ^]^ etc, which is a promising strategy to realize practical molecular switches and logic gates. In our supramolecular system, the azobenzene moiety can be switched selectively from *trans* to *cis* forms through UV irradiation, and reversely from *cis* to *trans* forms through visible irradiation (Figure 1b).^[^ ^32^, ^33^, ^34^ ^]^ *Trans*‐AzoC10 could be well‐recognized by PM‐*α*‐CD due to hydrophobic and van der Waals interactions between the inner surface of the CD cavity and hydrophobic guests. However, when *trans*‐AzoC10 is transformed to *cis*‐AzoC10, the bulky *cis*‐azobenzene moiety cannot be included into PM‐*α*‐CD anymore because of the size mismatch. By taking advantage of GMG‐SMJs, real‐time electrical measurements enabled us to analyze and even control this dynamic binding motion between a cavity PM‐*α*‐CD and a photoswitchable axle AzoC10, reaching the ultimate limit of analytical chemistry—single‐molecule or single‐event sensitivity.

## 2 Results and Discussion

The single‐molecule devices were constructed as reported in our previous work,^[^ ^35^ ^]^ including the fabrication of graphene field‐effect transistor (FET) as well as the formation of graphene point contacts. The edges of graphene point contacts were modified with abundant carboxyl groups due to the etching of oxygen plasma during the preparation process. Through a one‐step reaction, the molecular wires with amino terminals were covalently bridged to graphene electrodes with the fixed nanogap (≈2 nm) to form stable GMG‐SMJs. Detailed molecular synthetic routes and GMG‐SMJs device preparation steps are described in Figures S1−S3 (Supporting Information).

The current‐voltage (*I*−*V*) curves were used as evidence to verify the successful formation of GMG‐SMJs. As shown in Figure S4 (Supporting Information), the disconnected electrode pair exhibited an open circuit with conductance close to zero, while the electrode pair with molecular connection exhibited a typical molecular *I*−*V* curve under low bias voltages. Through precise control of the electrode gaps, 20 out of 154 devices showed the recovered molecular conductance and the connection yield was about 13%. The statistical analysis based on binomial distribution confirmed that the probability of single‐molecule connection was ≈94% (see the Supporting Information), which means that the transport behavior of GMG‐SMJs in the following measurements was mainly derived from the behavior of a single molecule.

To monitor the stochastic motion of photoswitchable single supramolecules, in‐situ, real‐time electric recordings were conducted on GMG‐SMJs at a source‐drain bias (*V*_D_) of 300 mV with high‐speed sampling frequency [57600 samples (Sa)/s]. The surface of the devices was covered with a home‐made polydimethylsiloxane microchannel filled with 1 × 10^−8^ M AzoC10 aqueous solution and the temperature of the solution was precisely controlled at 303 K. In addition, the solution was irradiated with sufficient visible (450 nm) or UV (365 nm) light for 30 min to ensure that all AzoC10 molecules are in the *trans* form or the *cis* form before each conductance measurement. Then, the measurements were performed under Vis/UV irradiation.

**Figure 2:**
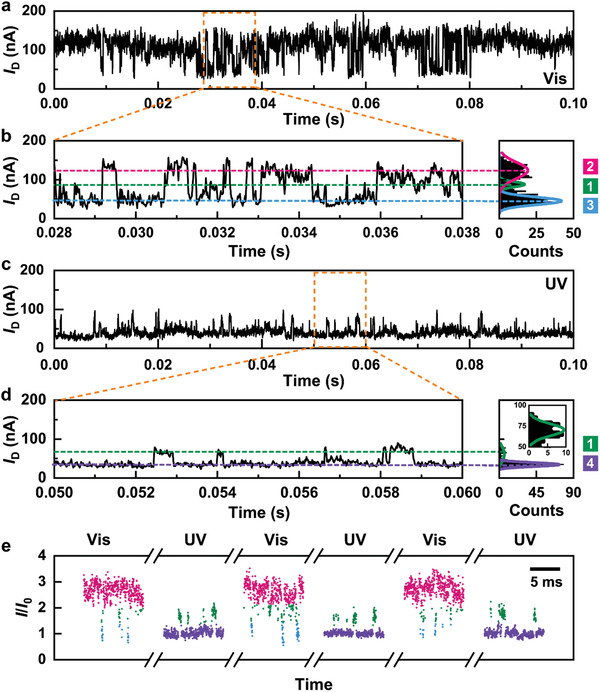
In‐situ, real‐time electrical recordings of the photoswitchable single supramolecule. a−d) *I*−*t* curves of the reversible binding process during 0.1 s and the corresponding enlarged parts measured in a water solution containing 1 × 10^−8^ M AzoC10 at 303 K under a, b) visible (450 nm) and c, d) UV (365 nm) irradiation. The right panels in b) and d) are the corresponding histograms of current values, showing trimodel and bimodal current distributions, respectively. *V*_D_ = 300 mV, Sampling rate = 57.6 kSa/s. e) Current oscillation signals of the single supramolecul in three cycles of interconversion between visible and UV irradiation. The colors of signals correspond to the current distribution in b) and d). All conductance measurements were conducted after sufficient visible or UV irradiation (30 min) to ensure that all AzoC10 molecules were in the *trans* form or the *cis* form.

As demonstrated in **Figure** **2**a–d, the current‐time (*I*−*t*) curves of GMG‐SMJs displayed a series of random telegraph signals. Under visible light irradiation, the resulting current‐count histogram revealed a trimodal Gaussian distribution centered at 45.4 ± 0.7 nA, 88 ± 1.0 nA and 123.8 ± 1.7 nA, respectively, indicating the existence of three distinct microstates during the reversible association and dissociation processes between the host PM‐*α*‐CD and the guest *trans*‐AzoC10 at the device‐liquid interface. Interestingly, after UV irradiation, the predominant high‐conductivity state disappeared and the original trimodal current‐count histogram was transformed into a bimodal Gaussian distribution centered at 34.5 ± 0.2 nA and 71.4 ± 8.2 nA, which could be related to the photoisomerization of the Azo unit in the guest molecule from the *trans* form to the *cis* form. In order to further verify the photo‐switching processes, three‐cycle conductance measurements of sequential visible and UV irradiations were carried out on the same GMG‐SMJs. The current oscillation signals were presented as *I*/*I*_0_, where *I* refers to the current value and *I*_0_ refers to the lowest Gaussian‐fitted peak value in each period. We categorized the dots into four groups labelled with different colors, corresponding to different conductance states in the distribution. We found that the signals switched back‐and‐forth between three‐level fluctuations and two‐level fluctuations during three cycles, and the *I*/*I*_0_ value of each conductivity state remained basically unchanged, which exhibited the high reproducibility and stability of our GMG‐SMJ platform. Besides, systematic control experiments were conducted on the GMG‐SMJs in pure water or a 1 × 10^−8^ M pyridine hydrochloride solution (Figure S5 and Figure S6, Supporting Information) and the GMG‐SMJs without PM‐*α*‐CD connected to the side chain or a graphene ribbon device (partially‐cut) in a 1 × 10^−8^ M AzoC10 aqueous solution (Figure S7 and Figure S8, Supporting Information) under the same illumination conditions. There was no obvious fluctuation observed in these conductance measurements, which confirms that the previous regular switching fluctuations essentially originate from the host‐guest interaction between PM‐*α*‐CD and AzoC10, or the relative binding motion between them.

**Figure 3:**
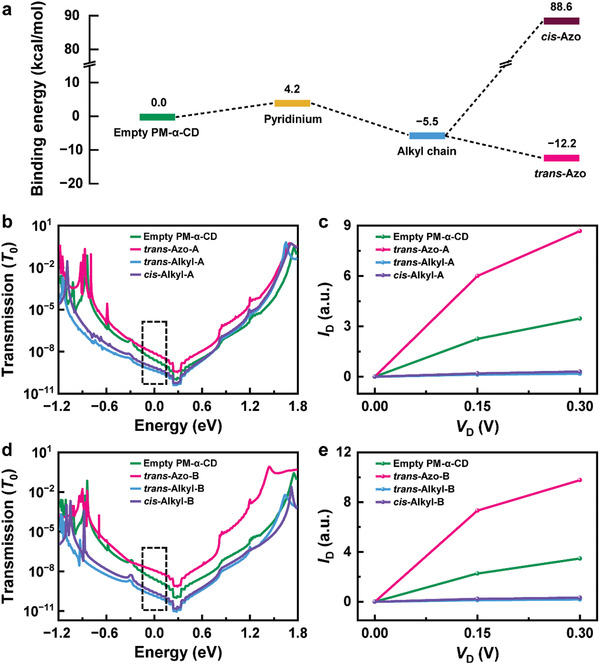
Theoretical analyses for the binding motion. a) Simulated energy profile of the binding states. The transmission spectra at zero bias and the calculated *I*−*V* curves of GMG‐SMJs under four different configurations in b, c) Mode A and d, e) Mode B, and the dashed boxes in b) and d) represent the bias window of 0.3 V.

To better understand the correlation between the conductance states and binding states of the host‐guest system, the molecular electronic structures and quantum transport properties were theoretically analyzed. We calculated the binding energies between the host PM‐*α*‐CD and the guest molecular units based on the following Equation (1) using the implicit solvent model (Figure S9, Supporting Information):

(1) $$E_{\mathrm{binding}}=G_{\mathrm{binding}-\mathrm{system}}\;-\left(G_{\mathrm{molecular}-\mathrm{unit}}+G_{\mathrm{PM}-\alpha -\mathrm{CD}}\right)$$

where *G* refers to the Gibbs free energy of the corresponding structure. The results showed that the binding energies of the *trans*‐azo moiety and the alkyl chain were negative, while the binding energies of the *cis*‐azo moiety and the pyridinium unit were positive (**Figure** **3**a). Therefore, the *trans*‐azo moiety and the alkyl chain can be well‐recognized by PM‐*α*‐CD during the dynamic process, which is consistent with the previous analysis. Furthermore, it should be noted that the binding energy of the *trans*‐azo moiety is lower than that of the alkyl chain, which means that the state of the *trans*‐azo moiety recognized by PM‐*α*‐CD dominates the binding process under visible irradiation. In combination with the experimental fact of the obvious high distribution for State 2 under visible irradiation and the disappearance of State 2 under UV irradiation, State 2 most likely corresponds to the case that the *trans*‐azo moiety is included in PM‐*α*‐CD. State 3 (the moderate proportion under visible irradiation) and State 4 (the highest proportion under UV irradiation) may correspond to the cases where the alkyl chain is included in PM‐*α*‐CD under visible and UV irradiation, respectively, while State 1 corresponds to the empty PM‐*α*‐CD.

To further assign the conductance states of these binding states, we analyzed the charge transport properties of different molecular configurations in combination with graphene electrodes using the nonequilibrium Green's function based on density functional theory.^[^ ^36^, ^37^, ^38^ ^]^ There exists the possibility that the OPE molecules attached to only one graphene sheet could affect the conductance signals. Therefore, we calculated transmission eigenstates at the Fermi level and transmission spectra for the model consisting of only one two‐bound OPE molecule and the model consisting of a two‐bound OPE molecule and a one‐bound OPE molecule, respectively. The main distribution of the eigenstate on the two‐bound OPE molecule and the almost overlapping of the transmission spectra for the two models near the Fermi level indicate that the two‐bound OPE molecule is the dominant transmission channel (Figure S10a−c, Supporting Information). Therefore, in order to simplify the model, we only constructed the single‐molecule model for the following theoretical analysis. Since the two rims of PM‐*α*‐CD are slightly different (≈0.45 nm and ≈0.52 nm for the inner diameters, respectively), the molecular axle could shuttle through PM‐*α*‐CD in two directions, thus the binding motion is divided into two modes and corresponds to two sets of different molecular configurations. In Mode A, when the alkyl chain is included in PM‐*α*‐CD, the azobenzene moiety is closer to the wide rim of PM‐*α*‐CD and the pyridinium unit is closer to the narrow rim of that (Figure S11a, Supporting Information). The opposite situation is defined as Mode B, as displayed in Figure S11b (Supporting Information). As discussed above, each motion mode contains four possible binding states (empty PM‐*α*‐CD, *trans*‐Azo‐A or *trans*‐Azo‐B, *trans*‐Alkyl‐A or *trans*‐Alkyl‐B and *cis*‐Alkyl‐A or *cis*‐Alkyl‐B). We first constructed the GMG‐SMJ models of these binding states, and then implemented the simulated annealing process^[^ ^39^, ^40^ ^]^ to obtain configurations with the lowest potential under an explicit solvent environment, followed by relaxation of these models. Finally, the transport properties of these optimized models were analyzed in QuantumATK software (Figure S12, Supporting Information). As reflected by the transmission spectra in Figure 3b,d, the conductance contribution from the perturbed highest occupied molecular orbital (*p*‐HOMO) is dominant since the corresponding transmission peaks are closer to the Fermi level in comparison with those of the perturbed lowest unoccupied molecular orbital (*p*‐LUMO). The transmission spectra of these configurations are significantly different near the Fermi level of graphene electrodes, thus affording different conductance states at low bias. For both of the two motion modes, the proximity of the *p*‐HOMOs to the electrode Fermi level follows the same sequence: *trans*‐Azo, empty PM‐*α*‐CD, *cis*‐alkyl and *trans*‐alkyl, which would be the sequence of conductance for these molecular configurations. In addition, the *I*−*V* curves were also calculated based on the **Equation (2)** of Landauer formula:

(2) $$I\;=\frac{2e}{h}\;\int_{-eV/2}^{eV/2}T\left(E\right)\left[f_{L}\left(E+eV/2\right)-f_{R}\left(E-eV/2\right)\right]dE$$

where *V* represents the bias window described by the dashed boxes in the transmission spectra (Figure 3b,d). The calculated *I*−*V* curves verified the conductance sequence as well. Although we have attributed the conductance states to different binding states of the host‐guest system, we cannot further distinguish between two motion modes since both experimental and theoretical results show that the conductance values for the same binding state in two modes are quite close to each other. In addition, in order to exclude the impact of some non‐covalent interactions at a remote location from the junction, we calculated the conductivity of the GMG‐SMJs when the guest molecule is near the molecular bridge (Figure S13a,b, Supporting Information). The transmission spectra and *I−V* calculation results show that, no matter whether AzoC10 is close to PM‐*α*‐CD or the molecular backbone (≈3 Å), it does not have an obvious impact on the current signal in comparison with that caused by host‐guest interaction (Figure S13c,d, Supporting Information).

**Figure 4:**
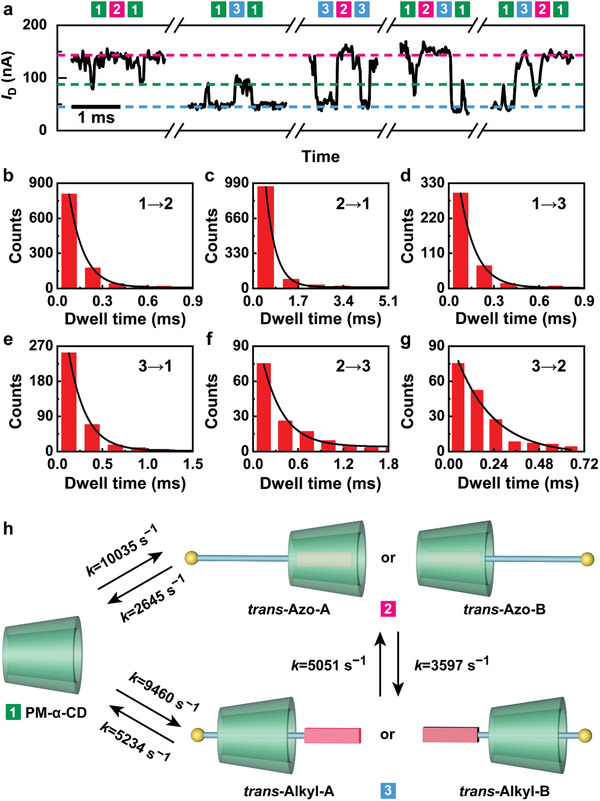
Kinetic analyses of *trans* isomers. a) *I*−*t* curves of five typical binding processes under visible irradiation. The first three are reciprocating motions and the latter two are unidirectional motions. b−g) Plots of time intervals of b) State 1 → State 2, c) State 2 → State 1, d) State 1 → State 3, e) State 3 → State 1, (f) State 2 → State 3 and (g) State 3 → State 2 from the same device at 303 K. h) Kinetic models for the binding processes in Mode A and Mode B.

Based on the assignment of all conductance states, detailed kinetic analyses were implemented. Under visible irradiation, the azobenzene moiety of AzoC10 was in the *trans* form and the binding motion was free and stochastic. **Figure** **4**a demonstrated the *I*−*t* curves of five typical binding processes. The first three trajectories represented the reciprocating motion: empty PM‐*α*‐CD (State 1) → *trans‐*Azo (State 2) → empty PM‐*α*‐CD (State 1), empty PM‐*α*‐CD (State 1) → *trans‐*Alkyl (State 3) → empty PM‐*α*‐CD (State 1) and *trans*‐Alkyl (State 3) → *trans*‐Azo (State 2) → *trans*‐Alkyl (State 3), respectively. The following two trajectories represented the unidirectional motion (both the starting state and the ending state were designated as empty PM‐*α*‐CD): empty PM‐*α*‐CD (State 1) → *trans‐*Azo (State 2) → *trans‐*Alkyl (State 3) → empty PM‐*α*‐CD (State 1) and PM‐*α*‐CD (State 1) → *trans‐*Alkyl (State 3) → *trans‐*Azo (State 2) → empty PM‐*α*‐CD (State 1). To obtain more kinetic information, the *I*−*t* curves were idealized into a three‐level interconversion from a segmental K‐means method based on a hidden Markov model analysis by using the QuB (Quantify Unknown Biophysics) software (Figure S14a, Supporting Information). Through statistical analysis, we found that the above‐mentioned binding processes occurred 965 times, 291 times, 121 times, 52 times, and 86 times within 1 s, respectively, of which the proportion of reciprocating motion is much larger than that of unidirectional motion. In the reciprocating motion, the number of cases for State 1 → State 2 → State 1 is three times greater than that of State 1 → State 3 → State 1, which was caused by the lower binding energy of *trans‐*Azo in comparison with *trans‐*Alkyl. Furthermore, the time intervals of all the conductance states were extracted from the idealized fitting according to the sequential relationship. Through single‐exponential fittings of the dwell time, the average lifetime of each state (*τ*) and the corresponding rate constant (*k* = 1/*τ*) could be obtained (Figure 4b−g, Table S1, Supporting Information). Assuming that the stochastic motion followed a simple Poisson process, *k*_1→2_, *k*_2→1_, *k*_1→3_, *k*_3→1_, *k*_2→3_ and *k*_3→2_ were fitted to be ≈10035 , ≈2645 , ≈9460 , ≈5234 , ≈3597 and, ≈5051 s^−1^, respectively (Figure 4h). It should be mentioned that *k*_1→2_>*k*_2→1_, *k*_1→3_>*k*_3→1_ and *k*_2→3_<*k*_3→2_, well corresponding to the thermodynamic stability sequence for the three states, *trans‐*Azo>*trans‐*Alkyl>PM‐*α*‐CD.

**Figure 5:**
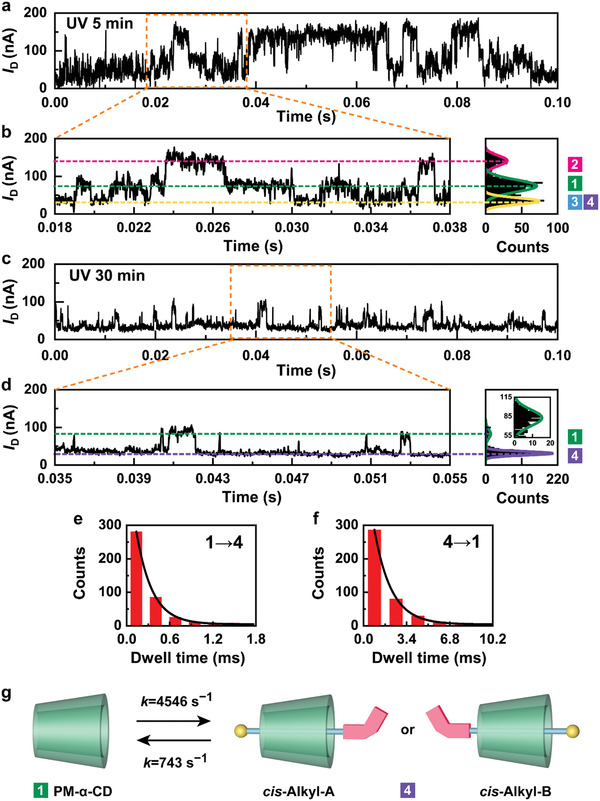
Kinetic analyses for *cis* isomers. *I*−*t* curves of the reversible binding process, the corresponding enlarged parts and the histograms of the current distributions under a, b) 5 min and c, d) 30 min UV irradiation. e, f) Plots of time intervals of e) State 1 → State 4 and f) State 4 → State 1 from the same device at 303 K. g) Kinetic models for the binding processes in Mode A and Mode B.

As the UV irradiation time increased, *trans*‐AzoC10 gradually photoisomerized into *cis*‐AzoC10. After the solution was exposed to UV light for 5 min, we observed that the proportion of *trans‐*Azo (State 2) dropped progressively and no new conductance state appeared (**Figure** **5**a,b). At that time, the lowest conductance state was considered as a mixed result of *trans‐*Alkyl (State 3) and *cis‐*Alkyl (State 4), which were difficult to distinguish, due to the incomplete photoisomerization conversion. However, after saturated UV irradiation for 30 min, *trans‐*Azo (State 2) and *trans‐*Alkyl (State 3) disappered completely and the *I*−*t* curves displayed a bimodal distribution of PM‐*α*‐CD (State 1) and *cis‐*Alkyl (State 4) (Figure 5c,d), revealing that the molecular motion changed from bidirectional shuttling to unidirectional oscillation. The kinetic data were also extracted and calculated following the same kinetic analysis method as before (Figure 5e,f, Figure S14b and Table S1, Supporting Information). The binding rates of *cis‐*AzoC10 into and out of empty PM‐*α*‐CD were found to be ≈4546 s^−1^ and ≈743 s^−1^, showing *cis‐*Alkyl is more thermodynamically stable than empty PM‐*α*‐CD (Figure 5g), which follows what we expected and is consistent with the previous studies.^[^ ^26^, ^27^ ^]^

## 3 Conclusion

In summary, this work demonstrated a reliable single‐molecule electrical method to monitor the stochastic motion of photoswitchable single supramolecules in real time, based on the host‐guest interaction between PM‐*α*‐CD and AzoC10. The high temporal resolution and single‐event sensitivity of GMG‐SMJs enabled us to directly observe the different motion phenomenon in the *trans* form or the *cis* form of AzoC10 and obtain the kinetic parameters during these processes, which are difficult to realize for the conventional ensemble characterization techniques. Moreover, we could reversibly switch the motion modes by irradiating visible and UV lights. This method provides a facile and effective tool for visualizing the dynamic process in stimulus‐responsive supermolecular systems at the single‐molecule level, which is of great significance for investigating the operation mechanisms of more complex systems such as ratchet mechanism in molecular machines.^[^ ^41^, ^42^, ^43^ ^]^ In addition, the introduction of stimulus‐responsive functional units on the guest molecules can inspire us to design and fabricate novel single‐molecule functional devices.

## 4 Experimental Section

### Molecular Synthesis

The details of molecular synthesis are provided in the Supporting Information.

### Device Fabrication and Molecular Connection

The process of fabricating graphene FET and graphene point contacts was described in detail in Figure S2 (Supporting Information). Single‐layer graphene was grown on copper foils through low‐pressure chemical vapor deposition and transferred to a 1.5 cm × 1.5 cm precleaned silicon wafer with a 300‐nm layer of thermally grown silicon oxide (SiO_2_) on the surface through the wetting transfer method. Next, gold marks were deposited on the corns of the wafer and a 40‐µm‐wide graphene strip was formed in the center of the wafer through photolithography, thermal evaporation and oxygen plasma etching. Finally, metallic electrodes arrays (8 nm Cr/80 nm Au) and passivation layer (40 nm SiO_2_) were respectively deposited on the wafer by photolithography, thermal evaporation and electron beam evaporation. The prepared graphene FET was spin‐coated with polymethyl methacrylate as the mask and etched using dash‐line lithography through high‐resolution electron beam lithography. Through fine oxygen plasma etching of the exposed window, carboxylic acid–functionalized graphene point contact arrays were finally formed.

For the molecular connection, the PM‐*α*‐CD functionalized molecule or control molecule was first dissolved in anhydrous pyridinium with a concentration of about 10^−4^ M. Then, the fresh devices and 1‐ethyl‐3‐(3‐dimethylaminopropyl) carbodiimide hydrochloride, a common dehydrating/activating agent, were added to the previously prepared solution (Figure S3, Supporting Information). After reacting in Ar for 48 h, the devices were taken out from solution, washed with acetone and ultrapure water, and dried with N^2^ gas stream. The PM‐*α*‐CD functionalized molecule or the control molecule was finally bridged between the graphene electrode pair with amide bonds.

### Electrical Characterization

The *I−V* tests were carried out carefully at room temperature in the ambient atmosphere through an Agilent 4155C semiconductor parameter system (direct current measurements) and a Karl Suss (PM5) manual probe station. The *I−t* curves were obtained by a locked‐in amplifier (HF2LI, Zurich Instruments Ltd.) with a low‐noise current preamplifier (DL1211) at a sampling rate of 57.6 kHz. A hot and cold chuck (HCC214S, INSTEC) equipped with a proportional‐integration‐differentiation control system and a liquid N_2_ cooling system was used to control the temperature of the solvent reservoir. When thermal equilibrium was reached (≈10 min) at particular temperatures, the *I−t* curves of GMG‐SMJs were recorded.

### Statistical Analysis

The QuB software was used to idealize the collected current data based on the hidden Markov model. The number of total events and the dwell time of each signal event were extracted after the idealization. The dwell time was then fitted to a single‐exponential decay function and the average lifetime was generated using Origin 2020b. Data were expressed as mean ± standard deviation (SD).

## Conflict of Interest

The authors declare no conflict of interest.

## Supporting information

Supporting Information

## Data Availability

The data that support the findings of this study are available from the corresponding author upon reasonable request.
