# Supplementary material for: Stochastic Binding Dynamics of a Photoswitchable Single Supramolecular Complex
Source: Adv Sci (Weinh). 2022 Mar 2;9(13):2200022. doi: 10.1002/advs.202200022 (PMC9069358; doi:10.1002/advs.202200022)
Supplement: Supplementary file 1 — Supporting Information [file ADVS-9-2200022-s001.pdf]

ADVANCED SCIENCE
Open Access

Supporting Information

for *Adv. Sci.*, DOI 10.1002/advs.202200022

Stochastic Binding Dynamics of a Photoswitchable Single Supramolecular Complex

Dingkai Su, Shuyao Zhou, Hiroshi Masai, Zihao Liu, Ce Zhou, Chen Yang, Zhizhou Li, Susumu Tsuda, Zhirong Liu, Jun Terao* and Xuefeng Guo**

ADVANCED SCIENCE
Open Access

Supporting Information

for *Adv. Sci.,* DOI: 10.1002/advs.202200022

Stochastic Binding Dynamics of a Photoswitchable Single Supramolecular Complex

Dingkai Su, Shuyao Zhou, Hiroshi Masai, Zihao Liu, Ce Zhou, Chen Yang, Zhizhou Li, Susumu Tsuda, Zhirong Liu, Jun Terao* and Xuefeng Guo**

Supporting Information for

Stochastic Binding Dynamics of a Photoswitchable Single Supramolecular Complex

Dingkai Su, Shuyao Zhou, Hiroshi Masai, Zihao Liu, Ce Zhou, Chen Yang, Zhizhou Li, Susumu Tsuda, Zhirong Liu, Jun Terao* and Xuefeng Guo**

D. Su†, S. Zhou†, Z. Liu, C. Zhou, C. Yang, Z. Li, Z. Liu, X. Guo
Beijing National Laboratory for Molecular Sciences, National Biomedical Imaging Center, College of Chemistry and Molecular Engineering
Peking University
Beijing 100871, P. R. China
E-mail: guoxf@pku.edu.cn; LiuZhiRong@pku.edu.cn

H. Masai†, J. Terao
Department of Basic Science, Graduate School of Arts and Sciences
The University of Tokyo
Tokyo 153-8902, Japan
E-mail: cterao@mail.ecc.u-tokyo.ac.jp

S. Tsuda
Department of Chemistry
Osaka Dental University
Osaka 573-1121, Japan

X. Guo
Center of Single-Molecule Sciences, Institute of Modern Optics, Frontiers Science Center for New Organic Matter, College of Electronic Information and Optical Engineering
Nankai University
38 Tongyan Road, Jinnan District, Tianjin 300350, P. R. China

Table of Contents

1. **Molecular Synthesis and Characterization..S3**
2. **Device Fabrication and Molecular Connection……... S6**
3. **Statistical Analysis of Single-Molecule Connection Probability......................S7**
4. **Electrical Characterization and Control Experiments……………………….....S8**
5. **Theoretical Calculations..S11**
6. **Kinetic Analysis for the Single-Molecule Junctions..S16**
7. **References..S17**

1. Molecular Synthesis and Characterization

1.1 General Remarks

Material: Unless otherwise stated, commercially available chemicals were used as received. Diisopropylamine was degassed through N_2 bubbling before use. THF was purchased from Kanto Chemical and further purified by passage through activated alumina under positive argon pressure as described by Grubbs *et al.*[1] **S1**[2] and PM-α-CD[3] was prepared according to the previously reported procedures.

NMR Spectroscopy: ^{1}H NMR (500 MHz) and ^{13}C NMR (126 MHz) were measured with a Bruker AVANCE-500 spectrometer. The ^{1}H NMR chemical shifts are reported relative to tetramethylsilane (TMS, 0.00 ppm). The ^{13}C NMR chemical shifts are reported relative to $^{13}CDCl_3$ (77.16 ppm).

Mass Spectroscopy (MS): Electrospray ionization time-of-flight (ESI-TOF) mass spectra were obtained on a Waters Xevo G2-S Tof mass spectrometer.

Preparative Recycling Gel Permeation Chromatography (GPC): Preparative recycling GPC was performed with a JAI LC9140 System equipped with a JAIGEL-2.5H column, a JAI UV DETECTOR 310, and a JAI RI DETRCTOR RI-5 using $CHCl_3$ as the eluent at a flow rate of 14 mL min^{-1}.

1.2 Synthetic Procedure

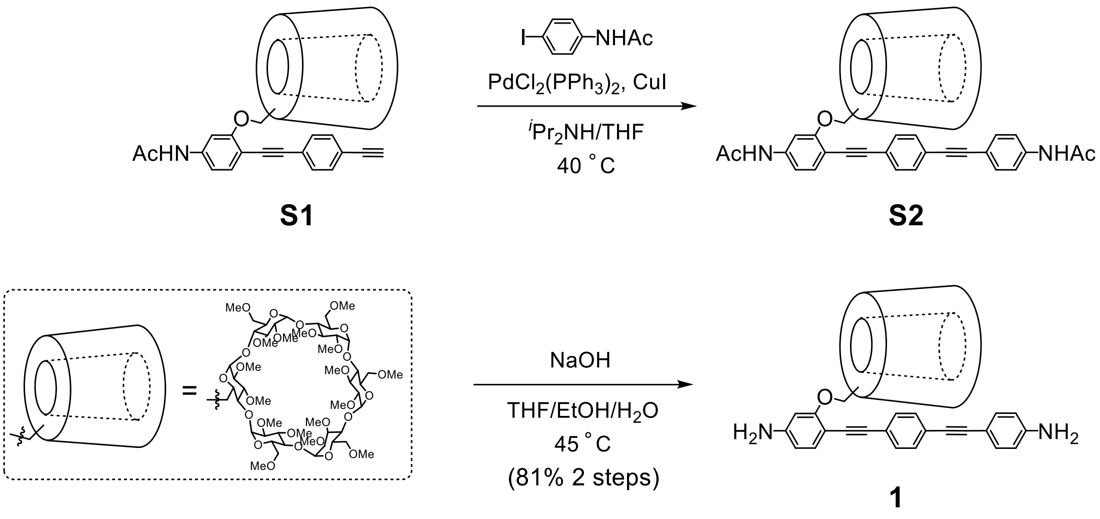

Figure S1. Schematic process of molecular synthesis.

Under a nitrogen atmosphere, **S1 (**200 mg, 136 μmol) and $PdCl_2$ $(PPh_3)_2$ (9.6 mg, 14 μmol) and CuI (1.3 mg, 6.8 μmol) were added into degassed diisopropylamine (10 mL) and THF (3 mL). *P*-iodoacetanilide (142 mg, 544 μmol) was added into the solution, and then the reaction mixture was stirred at 40 °C overnight. The mixture was dried *in vacuo*. The residue was purified by column chromatography on silica gel (1:1 toluene:EtOAc, 85:15 EtOAc:MeOH) to yield crude **S2** as a yellow solid (247 mg). Without further purification, **S2** was dissolved in THF (15 mL), EtOH (6 mL), and water (6 mL). NaOH (4.8 g, 120 mmol) was added into the solution. The reaction mixture was stirred at 45 °C for 2 d, and the mixture was diluted with water. The organic layer was extracted with $CHCl_3$ and Et_2O, and then dried with Na_2SO_4. The solvent was removed *in vacuo*, and the residue was purified by GPC with $CHCl_3$ as the eluent to yield **1** as a yellow solid (168 mg, total yield 81%).

ESI-TOF MS: (*m/z*) 1539.62 ($[M+Na^+]^+$, $C_{75}H_{108}N_2O_{30}Na$, calcd. 1539.69).

^1H NMR (500 MHz, $CDCl_3$, r.t.) : $_H$ = 7.50 (d, *J* = 8.2 Hz, 2H, ArH), 7.41 (d, *J* = 8.2 Hz, 2H, ArH), 7.34 (d, *J* = 8.3 Hz, 2H, ArH), 7.25 (d, *J* = 8.2 Hz, 1H, ArH), 6.64 (d, *J* = 8.3 Hz, 2H, ArH), 6.24 (d, *J* = 8.2 Hz, 1H, ArH), 6.20 (s, 1H ArH), 5.17-3.05 (m, 97H,

NH_2, CD-H, OCH_3).

^{13}C NMR (126 MHz, $CDCl_3$, r.t.) : $_C$ = δ 160.71, 148.38, 146.86, 134.39, 133.09, 131.28, 129.72, 128.79, 123.58, 122.83, 114.86, 112.58, 107.65, 102.99, 100.56, 100.41, 100.29, 100.23, 100.21, 99.76, 99.43, 91.75, 88.84, 87.54, 82.88, 82.74, 82.65, 82.62, 82.47, 82.38, 82.30 (peaks overlapped), 82.11, 81.33 (peaks overlapped), 81.26, 81.20, 71.88, 71.70, 71.62, 71.56, 71.51 (peaks overlapped), 71.42, 71.39, 71.37, 71.33, 70.58, 67.9, 62.03, 61.96 (peaks overlapped), 61.89, 59.32, 59.21 (peaks overlapped), 59.15, 59.09, 58.38, 58.04, 58.00, 57.95, 57.93, 57.53.

1.3 NMR Spectra

^{1}H NMR spectrum of **1**

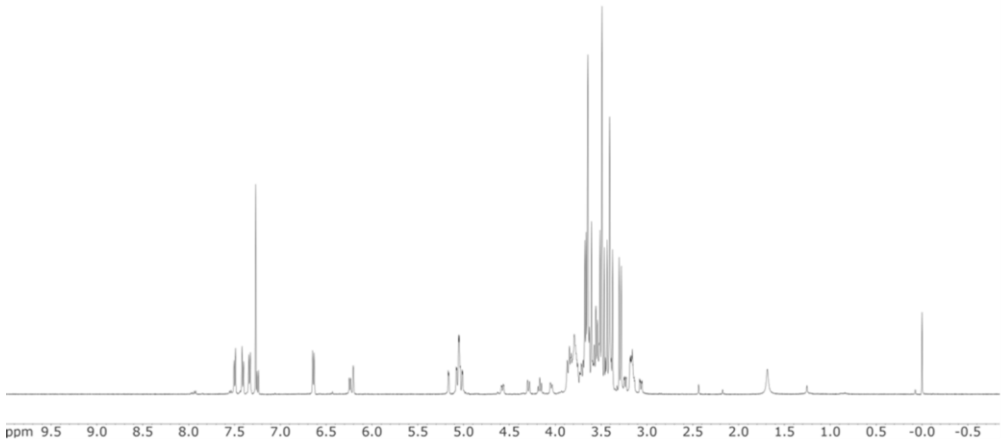

^{13}C NMR spectrum of **1**

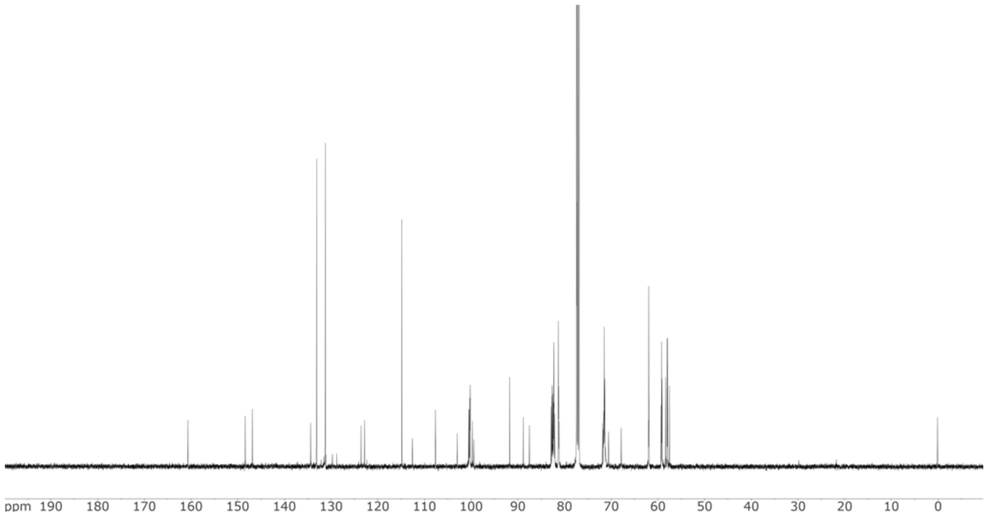

2. Device Fabrication and Molecular Connection

2.1 Device Fabrication

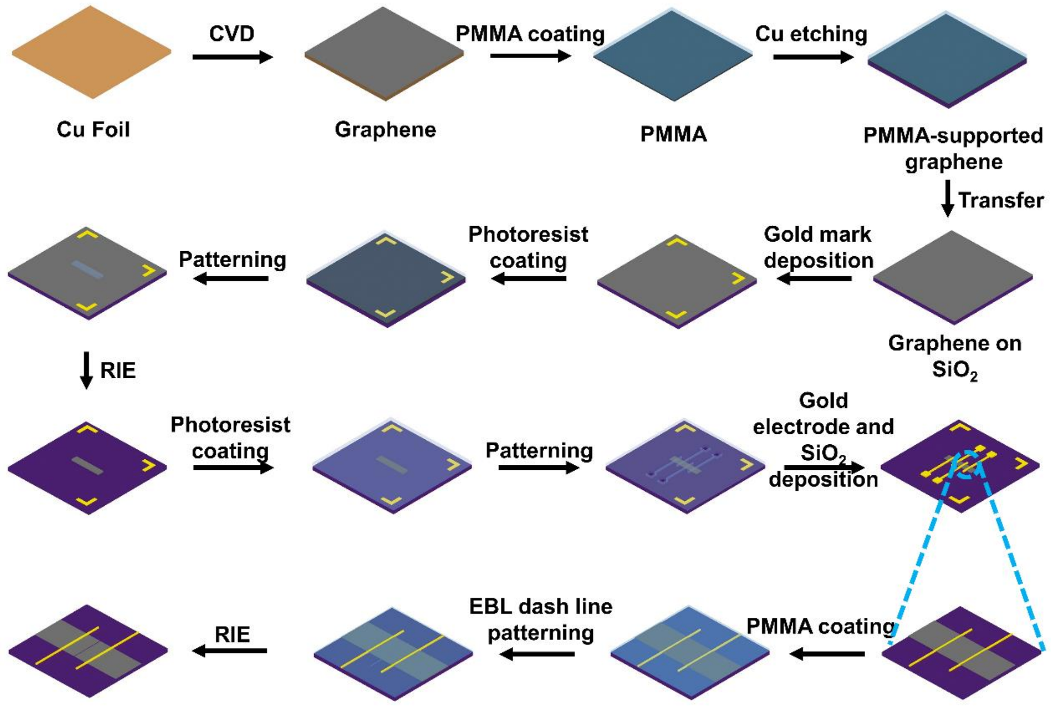

Figure S2. Schematic process of single-molecule device fabrication.

2.2 Molecular Connection

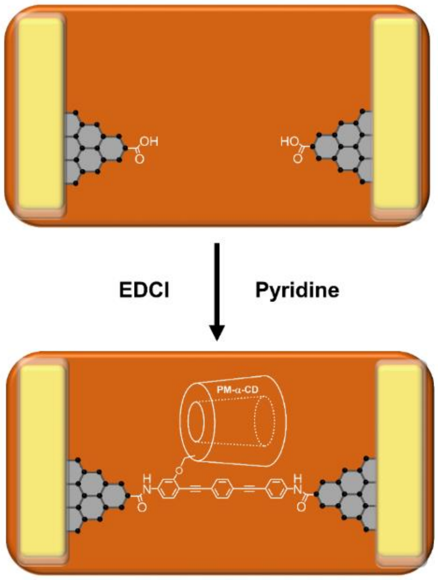

Figure S3. Schematic of one-step molecular connection to nanogapped graphene electrodes.

3. Statistical Analysis of Single-Molecule Connection Probability

There were m = 210 pairs of graphene point contacts between adjacent gold electrodes. Under optimized conditions, the probability of molecular connection for a pair of gold electrode reached ~13 % and the probability of molecular connection for each graphene point contact is p = 13%/210= 0.062%.

According to the binomial distribution, the probability of n molecules connected to a pair of gold electrodes (G_n) is expressed as:

$$G_n = \frac{m!}{n!\,(m-n)!} p^n (1-p)^{m-n}$$

The probability of molecules connected to a pair of gold electrodes ($Y_{connection}$) is expressed as:

$$Y_{connection} = 1 - G_0 = 1 - \frac{m!}{0!\,(m-0)!} p^0 (1-p)^m = 1 - (1-p)^m$$

Therefore, the probability of single-molecule connection is: $G1/Y$connection = ~94%.

This result shows that there is most likely only one molecular connection between a pair of successfully connected gold electrodes. Further experimental evidence for the successful formation of GMG-SMJs can be found in a recent work, where we used a self-built photoelectrical integrated characterization system to demonstrate the single-molecule connection.[4]

4. Electrical Characterization and Control Experiments

4.1 Electrical Characterization of PM-α-CD Based Devices

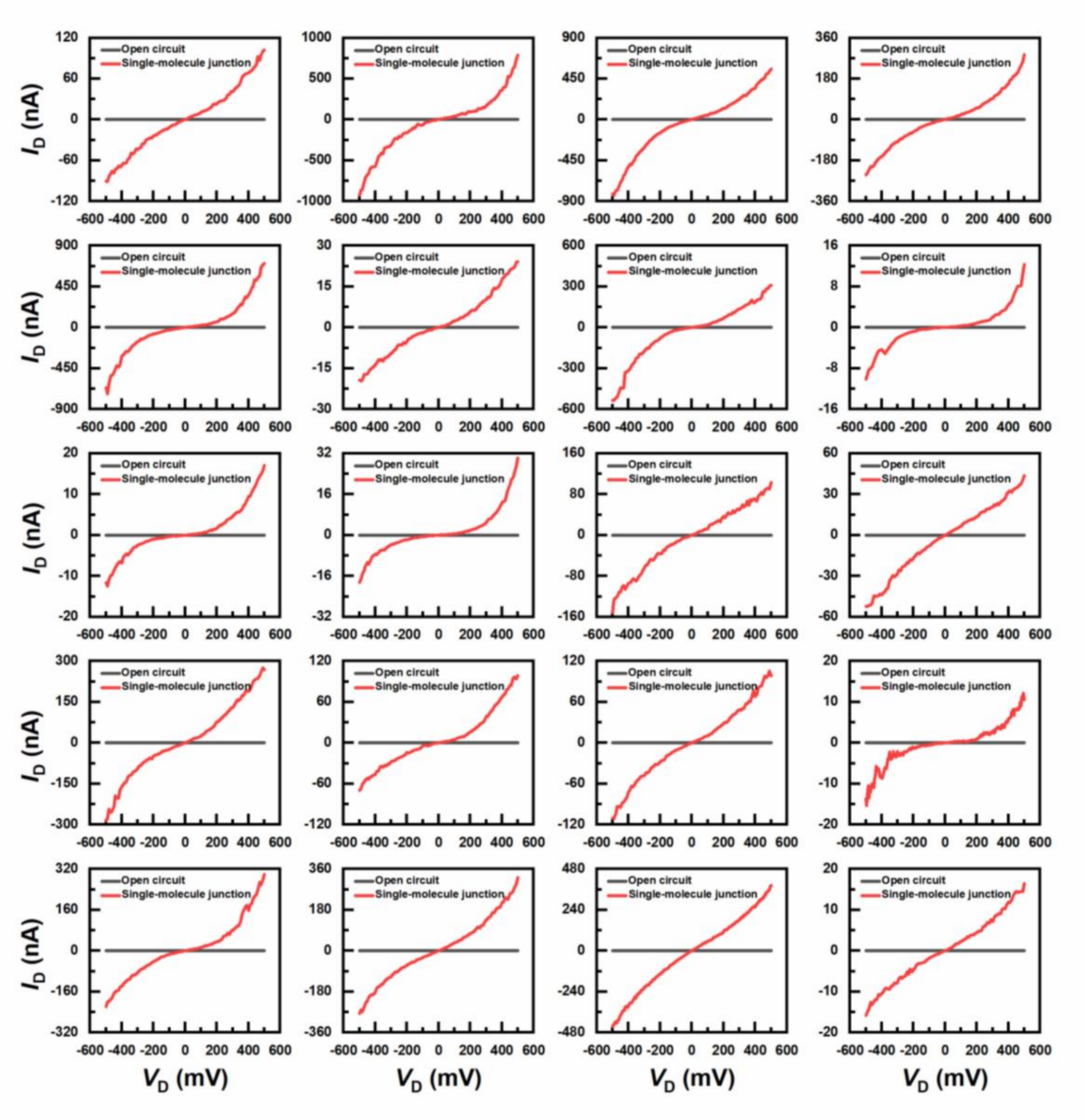

Figure S4. *I−V* curves of 20 typical PM-α-CD based GMG-SMJs in the solid state. Black lines represent open circuits and red lines represent the working GMG-SMJs.

4.2 Electrical Characterization of Control Devices

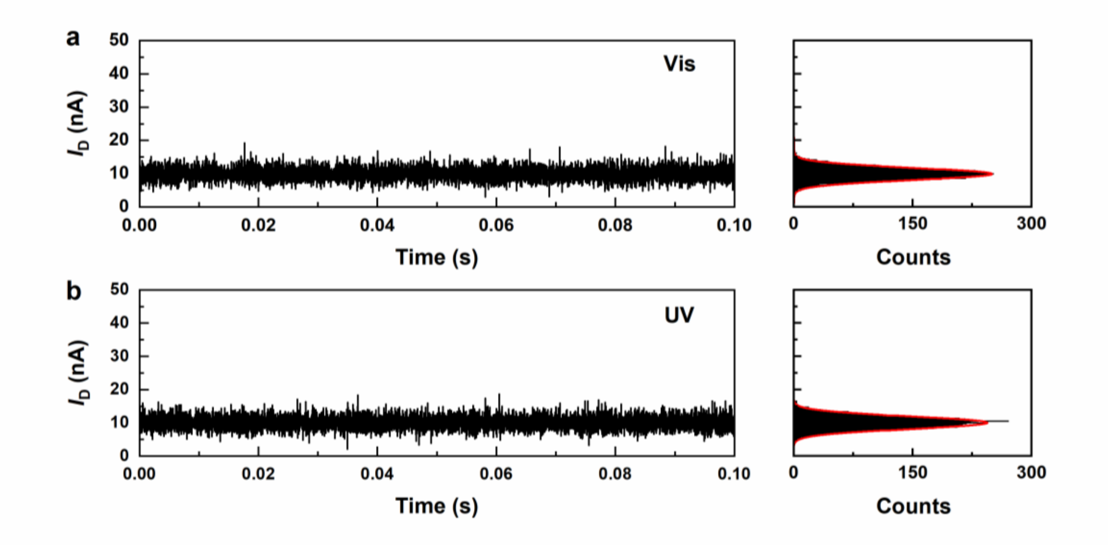

Figure S5. *I*–*t* curves of PM-α-CD based GMG-SMJs and the corresponding histograms of the current distributions measured in a pure water solution at 303 K under a) visible (450 nm) and b) UV (365 nm) irradiation. V_D = 300 mV, Sampling rate = 57.6 kSa/s.

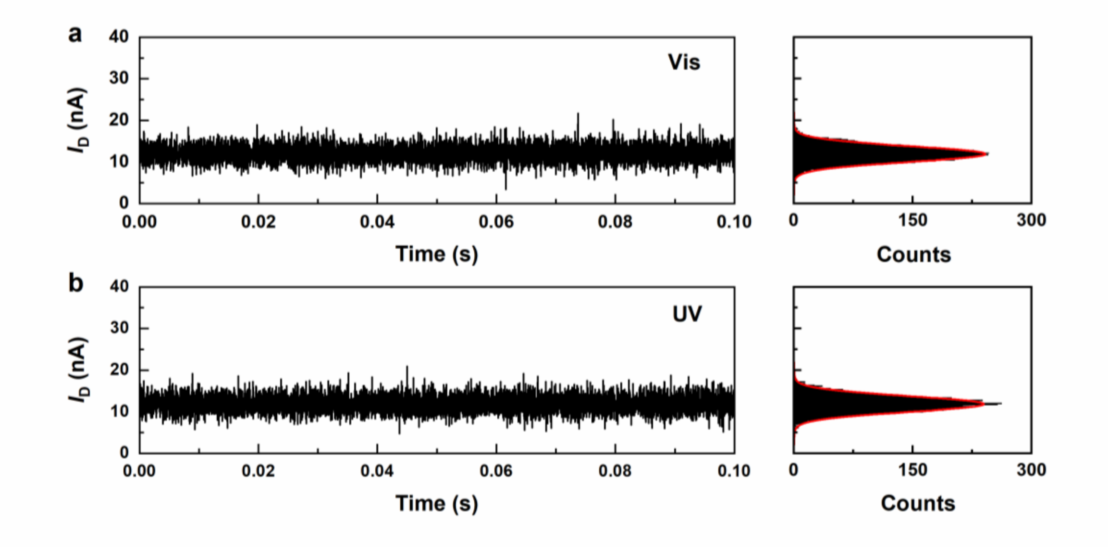

Figure S6. *I*–*t* curves of PM-α-CD based GMG-SMJs and the corresponding histograms of the current distributions measured in a 1×10^{-8} M pyridine hydrochloride aqueous solution at 303 K under a) visible (450 nm) and b) UV (365 nm) irradiation. V_D = 300 mV, Sampling rate = 57.6 kSa/s.

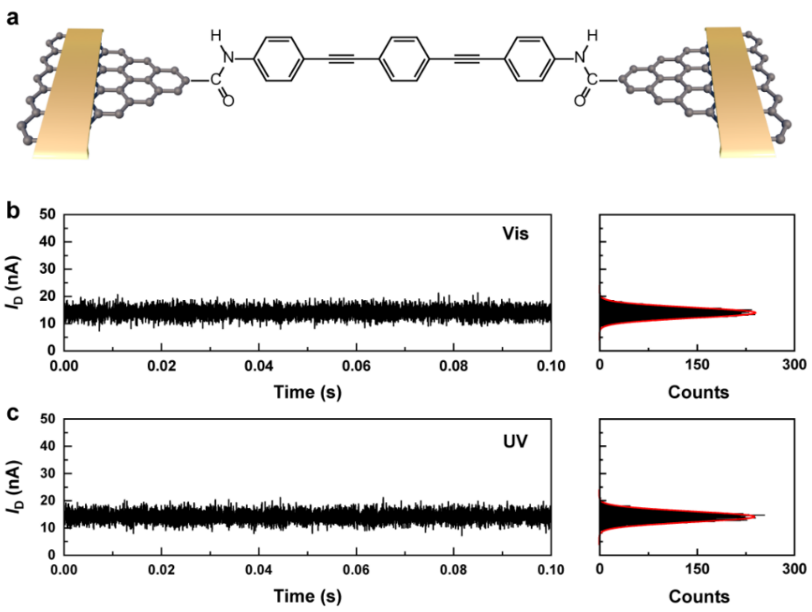

Figure S7. Device characteristics of control experiments. a) Device structure of GMG-SMJs with control molecules. b, c) *I*–*t* curves and the corresponding histograms of the current distributions measured in a 1 × 10^{-8} M AzoC10 aqueous solution at 303 K under (b) visible (450 nm) and (c) UV (365 nm) irradiation. V_D = 300 mV, Sampling rate = 57.6 kSa/s.

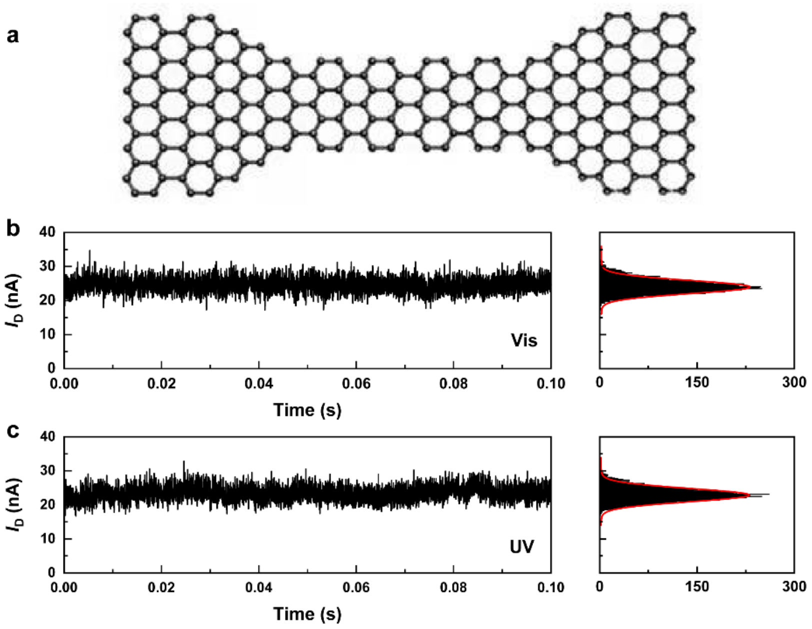

Figure S8. Device characteristics of control experiments. a) Device structure of a graphene ribbon device. b, c) *I*–*t* curves and the corresponding histograms of the current distributions measured in a 1 × 10^{-8} M AzoC10 aqueous solution at 303 K under (b) visible (450 nm) and (c) UV (365 nm) irradiation. V_D = 300 mV, Sampling rate = 57.6 kSa/s.

5. Theoretical Calculations

5.1 Binding Energy Calculations

The structures of molecular units, PM-α-CD and binding systems for the calculation of binding energies are shown in Figure S6, and each model of binding systems was constructed by inserting the molecular unit into PM-α-CD. The geometries of all the structures were optimized at the M062x/6-31G(d) level.[5] Water solvation was included during the optimizations by using the SMD solvation model.[6] Frequency calculations verified that all the structures have no imaginary frequency. All the calculations were performed with the Gaussian 16 software.[7]

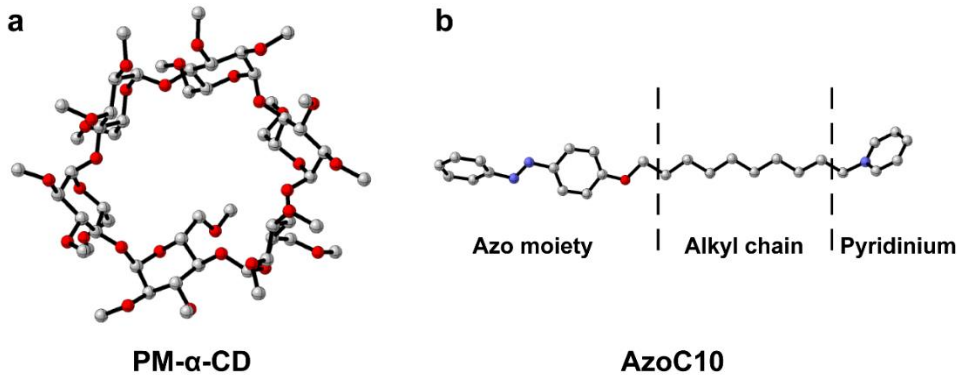

Figure S9. Models of a) PM-α-CD and b) Azo-C10. The C-H bonds were omitted for simplification.

5.2 Conductance Calculations

A two-probe system which connects a molecule with two graphene electrodes was constructed to model molecular junctions. Since the structures are flexible, the initial configurations of the junctions were generated by using a simulated annealing method in Gromacs program[8]. The junction models were parameterized using a general amber force field (gaff) with ACPYPE software[9], and the charge value for each atom of the guest molecule was replaced by the RESP charge value calculated at m062x/6-31g(d) level. To simulate the water solvent environment[10], the junction model was inserted into a 5×5×5 nm box filled with explicit water molecules, and the TIP4P-EW water model[11] was used with AMBER99SB force field. We set the peak temperature at 303 K, and the time length of each annealing cycle was set as 200 ps. Twenty annealing processes were implemented for each junction model, and the configuration with lowest potential was chosen for further energy minimization. Then, the fully relaxed molecular junction models were combined with two semi-infinite *p*-type doped graphene electrodes to construct the transport device models, and a bromonium anion was put near the molecular bridge as the counter ion. The transport behavior of devices was calculated by combining density functional theory (DFT) and non-equilibrium Green's functions method (NEGF) in the Atomistix toolkit (ATK) package. We adopted local density approximation (LDA) with a double-zeta plus polarization basis set. The density mesh cutoff was set to be 200 Rydberg. A *k*-point mesh of 2×1×111 and 12×1 was used for device self-consistent calculations and conductance analysis (transmission eigenstate, transmission spectra and $I-V$ curves) calculations, respectively. The source of the transmission eigenstate was set as left since the left electrode is the source electrode, and the isovalue was set as low as 0.000015 $Å^{-3/2}$.

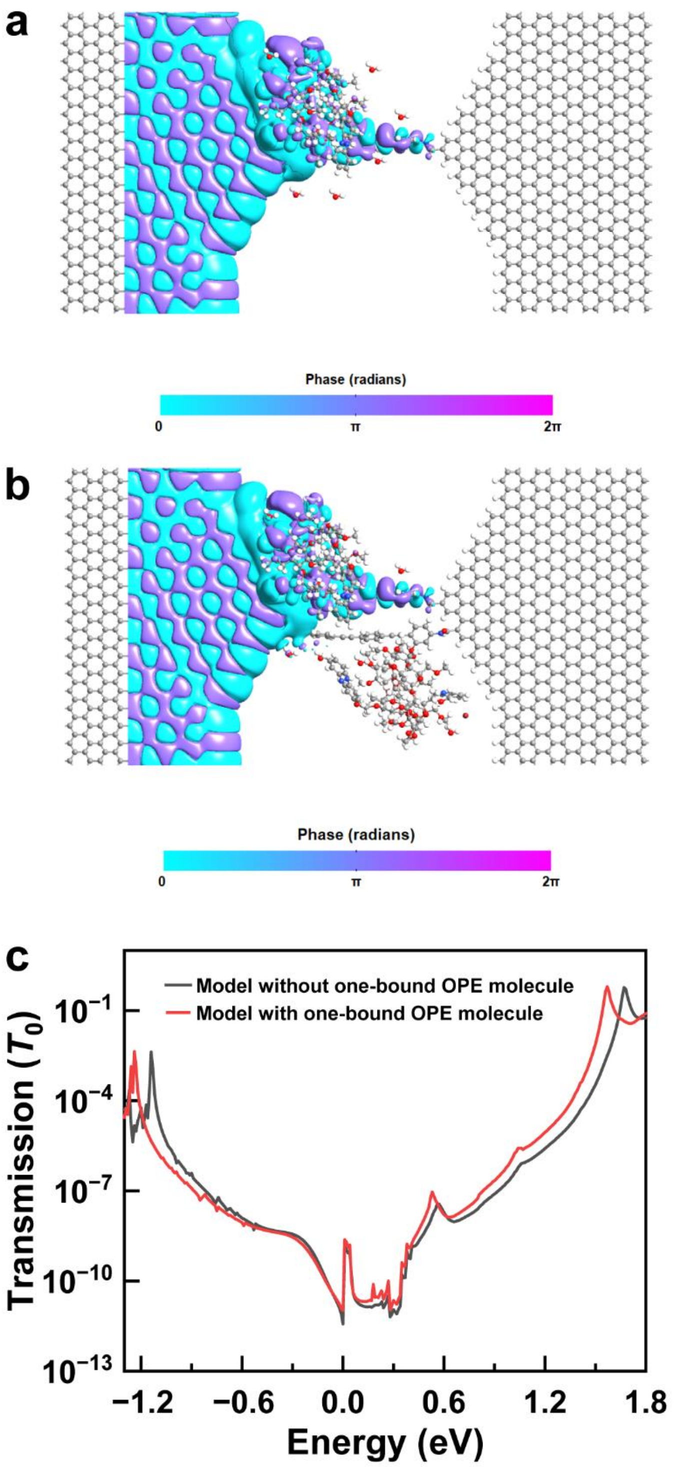

Figure S10. The transmission eigenstates at the Fermi level of the models of one OPE molecule spanning two sheets of graphene a) without or b) with one OPE molecule bound to one graphene sheet. The bias was set as 0 V. c) The transmission spectra at zero bias for the two models. AzoC10 guest molecules are bound to PM-α-CD for both of these two OPE molecules. To avoid mirror effect for the periodic system, extended electrodes were adopted for these two models.

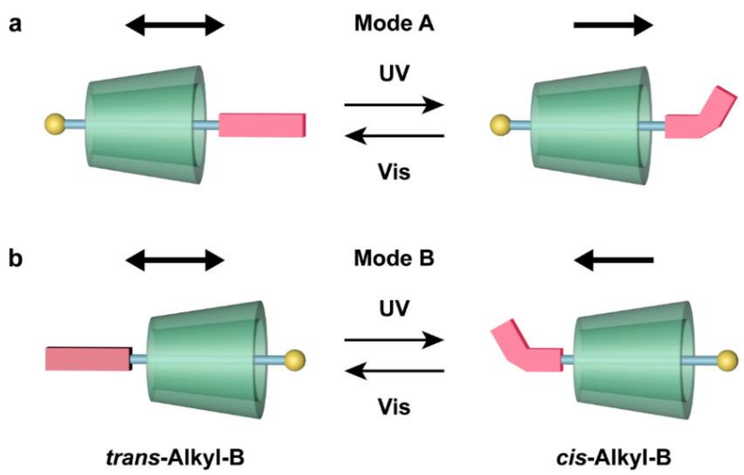

Figure S11. Schematic representation of two modes of the stochastic binding motion according to cases that the azobenzene moiety was closer to a) the wide rim (Mode A) or b) the narrow rim (Mode B) when the alkyl chain was included in PM-α-CD.

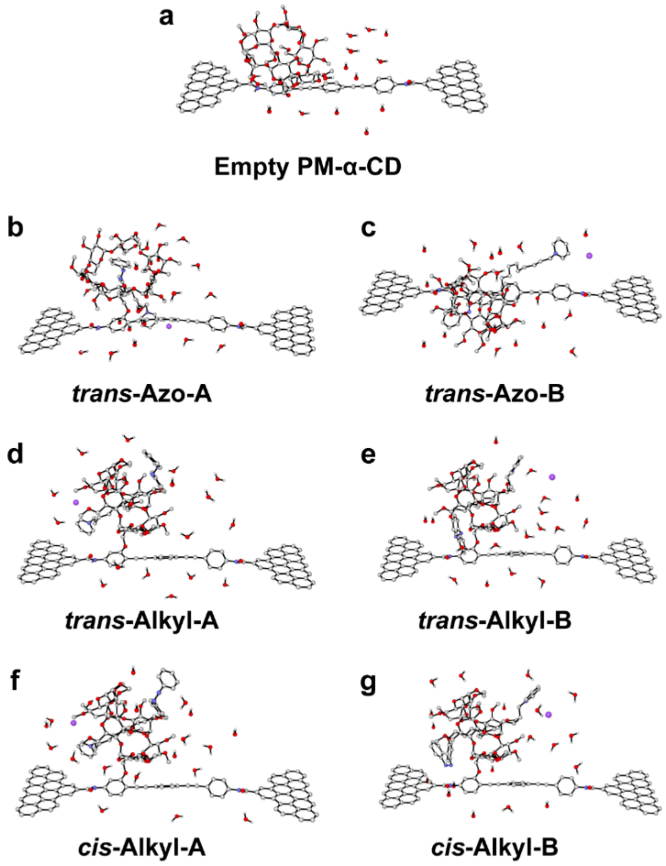

Figure S12. Typical molecular configurations of AzoC10 combined with PM-α-CD during shuttling processes: a) Empty PM-α-CD, b) *trans*-Azo-A, c) *trans*-Azo-B, d) *trans*-Alkyl-A, e) *trans*-Alkyl-B, f) *cis*-Alkyl-A and g) *cis*-Alkyl-B.

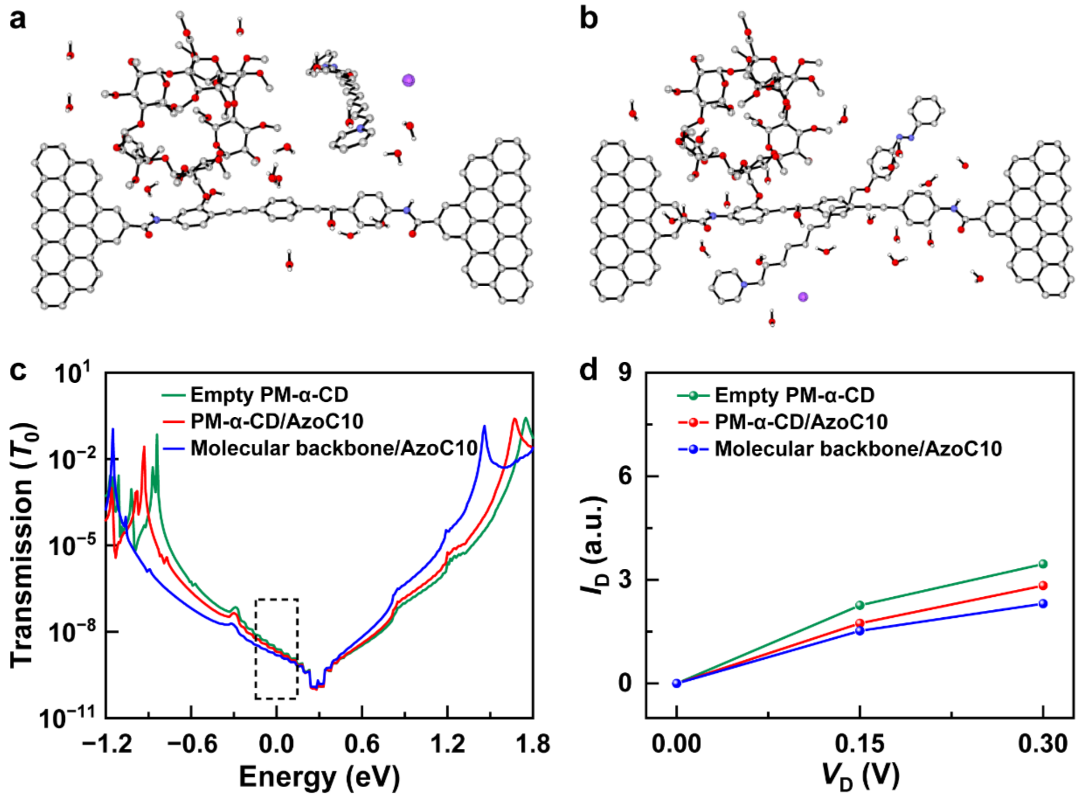

Figure S13. Typical molecular configurations of AzoC10 close to a) Empty PM-α-CD and b) molecular backbone. The comparison of Empty PM-α-CD and these two configurations in c) the transmission spectra at zero bias and d) the calculated $I-V$ curves, and the dashed box in (c) represents the bias window of 0.3 V.

6. Kinetic Analysis for the Single-Molecule Junction

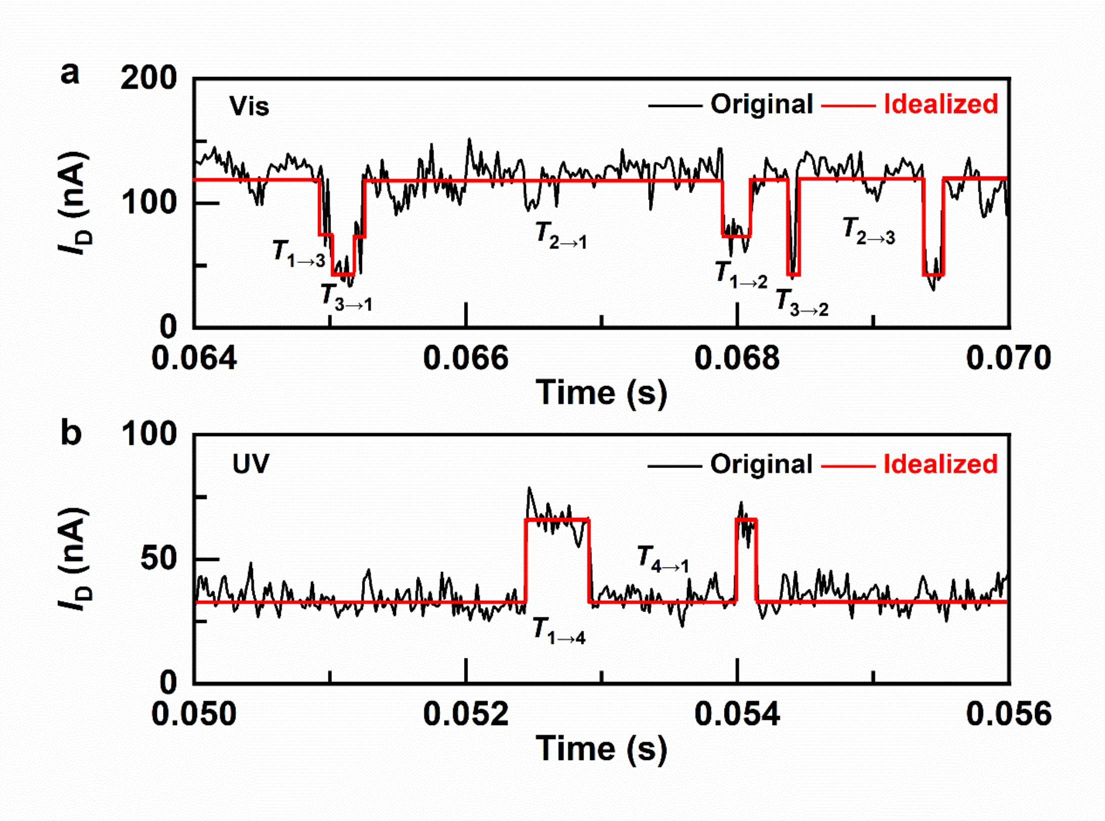

Figure S14. *I*–*t* curves (black) of PM-α-CD based GMG-SMJs immersed in the 1×10^{-8} M AzoC10 aqueous solution at 303 K and the idealized fit (red) obtained from a segmental K-means method based on hidden Markov model analysis by using the QUB software under a) visible (450 nm) and b) UV (365 nm) irradiation. V_D = 300 mV, Sampling rate = 57.6 kSa/s.

Table S1. The average lifetimes of each state (τ) in the shuttling process under different irradiation conditions.

τ [μs][a)]	Vis irradiation	UV irradiation
$\tau_{1\rightarrow2}$	99.65 ± 2.47	NA
$\tau_{2\rightarrow1}$	378.03 ± 20.35	NA
$\tau_{1\rightarrow3}$	105.71 ± 3.07	NA
$\tau_{3\rightarrow1}$	191.07 ± 5.64	NA
$\tau_{2\rightarrow3}$	277.98 ± 32.98	NA
$\tau_{3\rightarrow2}$	197.99 ± 46.18	NA
$\tau_{1\rightarrow4}$	NA	219.99 ± 7.24
$\tau_{4\rightarrow1}$	NA	1346.45 ± 49.20

a) Data representation mean ± SD, sample size n = 3.

7. References

[1] A. B. Pangborn, M. A. Giardello, R. H. Grubbs, R. K. Rosen, F. J. Timmers, *Organometallics* **1996**, *15*, 1518.

[2] J. Terao, A. Wadahama, T. Fujihara, Y. Tsuji, *Chem. Lett.* **2010**, *39*, 518.

[3] M. Morisue, S. Ueda, M. Kurasawa, M. Naito, Y. Kuroda, *J. Phys. Chem. A* **2012**, *116*, 5139.

[4] C. Yang, L. Zhang, C. Lu, S. Zhou, X. Li, Y. Li, Y. Yang, Y. Li, Z. Liu, J. Yang, K. N. Houk, F. Mo, X. Guo, *Nat. Nanotechnol.* **2021**, *16*, 1214.

[5] Y. Zhao, D. G. Truhlar, *Theor. Chem. Acc.* **2007**, *120*, 215.

[6] A. V. Marenich, C. J. Cramer, D. G. Truhlar, *J. Phys. Chem. B* **2009**, *113*, 6378.

[7] M. J. Frisch, G. W. Trucks, H. B. Schlegel, G. E. Scuseria, M. A. Robb, J. R. Cheeseman, G. Scalmani, V. Barone, G. A. Petersson, H. Nakatsuji, X. Li, M. Caricato, A. V. Marenich, J. Bloino, B. G. Janesko, R. Gomperts, B. Mennucci, H. P. Hratchian, J. V. Ortiz, A. F. Izmaylov, J. L. Sonnenberg, Williams, F. Ding, F. Lipparini, F. Egidi, J. Goings, B. Peng, A. Petrone, T. Henderson, D. Ranasinghe, V. G. Zakrzewski, J. Gao, N. Rega, G. Zheng, W. Liang, M. Hada, M. Ehara, K. Toyota, R. Fukuda, J. Hasegawa, M. Ishida, T. Nakajima, Y. Honda, O. Kitao, H. Nakai, T. Vreven, K. Throssell, J. A. Montgomery Jr., J. E. Peralta, F. Ogliaro, M. J. Bearpark, J. J. Heyd, E. N. Brothers, K. N. Kudin, V. N. Staroverov, T. A. Keith, R. Kobayashi, J. Normand, K. Raghavachari, A. P. Rendell, J. C. Burant, S. S. Iyengar, J. Tomasi, M. Cossi, J. M. Millam, M. Klene, C. Adamo, R. Cammi, J. W. Ochterski, R. L. Martin, K. Morokuma, O. Farkas,

J. B. Foresman, D. J. Fox, Wallingford, CT **2016**.
[8] M. J. Abraham, T. Murtola, R. Schulz, S. Páll, J. C. Smith, B. Hess, E. Lindahl, SoftwareX **2015**, *1-2*, 19.
[9] A. W. Sousa da Silva, W. F. Vranken, *BMC Research Notes* **2012**, *5*, 367.
[10] N. G. Sgourakis, M. Merced-Serrano, C. Boutsidis, P. Drineas, Z. Du, C. Wang, A. E. Garcia, *J. Mol. Biol.* **2011**, *405*, 570.
[11] H. W. Horn, W. C. Swope, J. W. Pitera, J. D. Madura, T. J. Dick, G. L. Hura, T. Head-Gordon, *J. Chem. Phys.* **2004**, *120*, 9665.
